# Techniques to Study Antigen-Specific B Cell Responses

**DOI:** 10.3389/fimmu.2019.01694

**Published:** 2019-07-24

**Authors:** Jim Boonyaratanakornkit, Justin J. Taylor

**Affiliations:** Vaccine and Infectious Disease Division, Fred Hutchinson Cancer Research Center, Seattle, WA, United States

**Keywords:** B cells, B lymphocyte subsets, humoral immune response, antigens, vaccines

## Abstract

Antibodies against foreign antigens are a critical component of the overall immune response and can facilitate pathogen clearance during a primary infection and also protect against subsequent infections. Dysregulation of the antibody response can lead to an autoimmune disease, malignancy, or enhanced infection. Since the experimental delineation of a distinct B cell lineage in 1965, various methods have been developed to understand antigen-specific B cell responses in the context of autoimmune diseases, primary immunodeficiencies, infection, and vaccination. In this review, we summarize the established techniques and discuss new and emerging technologies for probing the B cell response *in vitro* and *in vivo* by taking advantage of the specificity of B cell receptor (BCR)-associated and secreted antibodies. These include ELISPOT, flow cytometry, mass cytometry, and fluorescence microscopy to identify and/or isolate primary antigen-specific B cells. We also present our approach to identify rare antigen-specific B cells using magnetic enrichment followed by flow cytometry. Once these cells are isolated, *in vitro* proliferation assays and adoptive transfer experiments in mice can be used to further characterize antigen-specific B cell activation, function, and fate. Transgenic mouse models of B cells targeting model antigens and of B cell signaling have also significantly advanced our understanding of antigen-specific B cell responses *in vivo*.

## Introduction

In his Nobel lecture in 1908, Paul Ehrlich likened the antibody-antigen interaction to a lock and key. He reasoned that antitoxins (antibodies) contained in a solution in the serum of immunized animals must be identical to a cellular receptor “for a really well-made key will not open different locks at the same time” (1). It took almost five decades before immunofluorescence microscopy was used to confirm the cellular origin of antibodies (2). Major strides in the B cell and antibody field followed in the 1970s with the development of hybridoma technology to produce monoclonal antibodies and the discovery that somatic rearrangement during B cell differentiation was responsible for antibody diversification (3, 4). The subsequent explosion of available monoclonal antibodies led to revolutionary diagnostic, therapeutic, and research reagents to distinguish different types of immune cells (5). Together, these discoveries have allowed us to probe humoral immunity at the level of the antigen-specific B cell.

## Why Study B Cell Responses? Opportunities for Applying Techniques to Study Antigen-Specific B Cells

Methods to probe the antigen-specific B cell response have advanced our understanding of how to harness the remarkable breadth of the B cell repertoire and the exquisite specificity of the individual B cell in developing (1) vaccine candidates that elicit protective antibodies; (2) antibodies that prevent disease when given prophylactically; and (3) antibodies that can be given as therapy after the onset of disease. Many of the vaccines currently available were originally developed empirically either by inactivating, attenuating, or administering a subunit of the pathogen. However, vaccine development against pathogens that are traditionally difficult to vaccinate against may rely on a deeper investigation of the B cell response to the antigens exposed on the surface of these pathogens.

For HIV-1, the discovery of broadly neutralizing antibodies (bnAbs) that protect against infection across diverse viral isolates has intensified efforts to understand the developmental pathway of the rare B cells that produce these antibodies (6–9). Insights into the ontogeny of these rare B cells could allow the design of a step-wise vaccine regimen that stimulates the germ-line precursor to expand and mature to produce circulating bnAbs which could protect against HIV acquisition (10, 11). For RSV, stabilized versions of the fusion (F) protein in the pre-fusion conformation have led to insights in the B cell's response to infection and has generated potentially safer and more efficacious vaccine candidates (12, 13). Influenza also performs fusion through the stem region of the hemagglutinin protein, and the identification of B cells that target this relatively conserved site has spurred research on the development of a universal influenza vaccine (14–16). Like RSV, HIV, and influenza, the fusion proteins of EBV and CMV exist in a pre-fusion conformation, and stabilization in their pre-fusion states could greatly accelerate vaccine development against these pathogens (17–19). Rare memory B cells producing antibodies specific for the EBV fusion machinery have been isolated; these can neutralize both B cell and epithelial cell infection (20). A new paradigm in malaria vaccine development is also emerging with the discovery of IgM+ and IgD+ memory B cells targeting the Merozoite Surface Protein 1, that rapidly respond to malaria re-infection (21). Further, highly potent neutralizing antibodies targeting a novel and conserved site on the Circumsporozoite Protein have been isolated from B cells (22). Together, these examples demonstrate the importance of studying antigen-specific humoral responses to infectious diseases. The solutions to the crystal structures of surface proteins for a variety of pathogens, the conformational stabilization of these antigens, and the application of the methods summarized in this review, to probe antigen-specific B cell responses, have created new opportunities for systematic and rational vaccine design for HIV, RSV, EBV, malaria, and many other pathogens.

The study of B cell responses has not only informed vaccine design but has also advanced our understanding of antibody-mediated autoimmune diseases, such as rheumatoid arthritis and systemic lupus erythematosus (23, 24). Up to 20% of mature, naïve B cells have receptors with the capacity to bind self-antigens (25). Although these cells are potentially pathogenic, the deletion of B cells with high affinity to self-antigen through apoptosis, anergy of B cells with low affinity to self-antigen, and the absence of T cell help combine together to protect against autoimmune disease in mice (26). The study of autoantigen-specific B cells and a detailed analysis of B cell subsets with pathogenic potential in humans could lead to a better understanding of how to prevent and treat autoimmune diseases.

Although the term antigen-specific B cell is used throughout this mini-review to denote the analysis of B cells based on binding between the B cell receptor (BCR) and a specific antigen used as bait, it is important to keep in mind that BCRs within the polyclonal B cell repertoire exhibit a spectrum of polyreactivity. On one end of the spectrum, a highly polyreactive BCR is able to bind multiple structurally unrelated antigens with physiologically relevant affinities. The frequency of polyreactivity in the normal adult human B cell repertoire has been estimated to be 4% of naïve B cells, 23% of IgG+ memory B cells, and 26% of intestinal IgA+ and IgG+ plasmablasts (27–29). On the other end of the spectrum, a mono reactive BCR is activated only when it encounters a single cognate antigen. Although there are exceptions, the accumulation of somatic hypermutations within the variable regions of the BCR during the process of affinity maturation is generally thought to lead to increased affinity and specificity for the cognate antigen (30, 31).

## *Ex vivo* Methods to Identify Antigen-Specific Primary B Cells

Several general techniques are commonly used to identify antigen-specific B cells (Table 1). The B cell enzyme linked immunospot (ELISPOT) technique relies on the principle of capturing the secreted antibody in the vicinity of each cell. In the B cell ELISPOT, antibody secreting B cells (ASCs) present in a sample or differentiated *in vitro* are added to plates coated with the antigen of interest. Antigen-specific antibodies will bind in close proximity to the location of the individual B cells producing those antibodies. Enzyme or fluorescent labeled secondary antibodies are then used to visualize spots of antibody secretion and binding to plate-bound antigen at the location of the ASCs. Each spot corresponds to antibody produced from a single antigen-specific B cell and therefore the technique is extremely sensitive. Secondary antibodies conjugated to combinatorial colored beads can also be used to detect the antibodies secreted from individual B cells with the advantage of multiplexing the assay (32). One limitation of the assay is its requirement for antibody secretion by B cells thereby limiting the assay to only a subset of B cells in the repertoire, namely ASCs (33). Memory B cells can be stimulated *in vitro* to differentiate into ASCs prior to addition to the antigen-coated plate (34). Further, the antigen-specific B cells identified by ELISPOT are generally not available for downstream analysis.

**Table 1:** Summary of techniques for studying antigen-specific B cells.

**Technique**	**Advantages**	**Potential limitations**	References^*^
***EX VIVO*** **METHODS**			
ELISPOT	Sensitive; quantitative	(1) Requires antibody secretion; (2) cells generally not available for downstream analysis	(32–35)
Limiting dilution	Allows functional screening of monoclonal antibodies for binding and neutralization	(1) Laborious; (2) requires antibody secretion; (3) transcriptional profiling of original cell not possible if *in vitro* expansion is used to screen the supernatant for antigen specificity	(36–41)
Flow cytometry	(1) Detection of low affinity antigen-specific B cells; (2) characterization and downstream analysis of cells is possible; (3) magnetic enrichment can improve sensitivity	(1) Over-biotinylation can lead to aggregation; (2) potential for confounding by cells that bind the fluorochrome, streptavidin, or linkers; (3) antigens must be soluble, stable, and readily labeled	(12, 21, 26, 39, 42–61)
***IN VIVO*** **METHODS**			
Adoptive transfer	Allows fate mapping of cells in the context of antigen presenting cells and T cells	Laborious and often limited to monoclonal populations	(26, 62–64)
Microscopy	(1) Localizes cells in tissue; (2) laser capture allows downstream analysis; (3) real-time imaging in living tissue is possible with multiphoton microscopy	Relies on antigens that can be readily labeled	(2, 62, 63, 65–70)
BCR transgenic mice	Useful for analyzing B cell development and responses and T/B cell interactions	Laborious and costly to develop; monoclonal B cells may not represent diverse polyclonal populations	(71, 72) See also Table 2
**EMERGING METHODS**			
Mass cytometry	(1) High dimensional analysis with minimal spillover across parameters; (2) magnetic enrichment can improve sensitivity	Cells are unavailable for downstream analysis	(73, 74)
DNA barcoding	Simultaneous analysis of multiple antigen specificities with transcriptional profiling	Cost and computational complexity	(75, 76)

*BCR, B cell receptor*.

* *Selected examples of each technique can be found in the listed references*.

Limiting dilution is another technique that has been used to isolate antigen-specific B cells. In this approach, primary cells can be diluted serially until individual B cells are separated in microwell plates (36). The B cells can then be cultured and expanded *ex vivo* and/or immortalized using EBV such that each well contains a monoclonal antibody (3, 37, 38). Antigen-specific B cells can be selected by screening the culture supernatants for monoclonal antibodies that bind an antigen of interest. Although antibodies can be sequenced and cloned, the requirement for an *ex vivo* culture prior to selection precludes determination of the transcriptional profile of the original B cell in this approach. This technique can potentially be time-consuming and laborious, but the use of microfluidics and robotics has greatly improved the throughput for selecting antigen-specific B cells (39). Advances in single cell next generation sequencing technology have allowed high throughput transcriptional profiling and sequencing of paired immunoglobulin heavy and light chains (40). In this approach, antigen specificity can be tested after monoclonal antibodies are cloned and produced using the sequencing data. This method can be useful in identifying antigen-specific B cells that have undergone clonal expansion after vaccination or acute infection (41).

Flow cytometry is the most common method used for single cell analysis and isolation (39). Flow cytometry-based analysis of antigen-specific B cells is dependent on labeling antigen with a fluorescent tag to allow detection. Fluorochromes can either be attached covalently via chemical conjugation to the antigen, expressed as a recombinant fusion protein, or attached non-covalently by biotinylating the antigen. After biotinylation, fluorochrome-conjugated streptavidin is added to generate a labeled tetramer of the antigen. Biotinylation of the antigen at a ratio ≤1 biotin to 1 antigen is important, since each streptavidin has the potential to bind four biotins. If the ratio of biotin to antigen is >1:1, then clumping and precipitation of the antigen out of solution can occur as soon as streptavidin is added. Alternatively, site directed biotinylation can be accomplished by adding either an AviTag or BioEase tag to the recombinant antigen prior to expression (77, 78). When site-specific biotinylation is utilized, researchers must keep in mind that the tag may occlude an epitope from recognition by B cells which can be problematic for vaccine antigens. Further, for proteins that oligomerize, multiple tags may be incorporated, possibly resulting in aggregation.

Another important consideration is the potential for confounding by B cells in the repertoire that bind to the fluorochrome, streptavidin, or any linkers rather than to the antigen of interest. Binding between fluorochromes, linkers, or streptavidin and BCRs from humans and mice never exposed to these antigens are generally of low affinity, and these BCRs are generally expressed by naïve and potentially polyreactive B cells (62, 79, 80). Dual labeling, in which the same antigen is separately labeled with two different fluorochromes, can be used to identify double positive B cells and remove confounding by B cells that bind the fluorochrome (12, 42). However, even when tetramers are utilized for dual labeling, streptavidin-specific B cells will contaminate the double positive population. To fully remove confounding from the fluorochrome, streptavidin, and linkers, a “decoy” tetramer can be used to identify these contaminating B cells (21, 26). In this approach, the same fluorochrome used to identify antigen-specific B cells is conjugated to a different fluorochrome such that the emission spectrum is altered by fluorescence resonance energy transfer (FRET) (26). Decoy-binding B cells can therefore be excluded from the true antigen-specific B cells. Notably, it is critical to use the same source of fluorochrome conjugated streptavidin in the tetramer and decoy reagent, because conjugation methods, recombinant streptavidin, and protein fluorochromes like R-phycoerythrin vary enough from company to company to alter some of the epitopes available for B cells to bind.

One weakness of the flow cytometric approach is the reliance on antigens that can be readily conjugated to a fluorochrome or biotinylated. In addition to recombinant proteins and synthesized peptides, labeled polysaccharides, lipids, haptens, virus-like particles, and pseudo viruses have also been used to identify antigen-specific cells by flow cytometry (33, 43–59). Further, epitope-specific B cells have been identified by screening bacteriophage-displays or microarray peptide libraries with polyclonal antibodies targeting the native antigen to select conformational epitopes that can be fused to fluorescent proteins for use in flow cytometry (47, 60). With technologic advancements increasing the number of simultaneously measurable parameters, antigen-specific B cells can be further characterized by cell surface markers and intracellular staining. Additionally, the immunoglobulin capture assay is a flow cytometry-based adaptation of the ELISPOT assay in which a streptavidin-conjugated anti-CD45 antibody carrying four biotinylated anti-IgG antibodies is used to simultaneously bind plasmablasts and capture secreted antibody followed by fluorescent-labeled antigen to detect antigen-specific plasmablasts (61). The mean fluorescence intensity measured by flow cytometry and normalized to the level of BCR expression also provides a measure of the relative amount of antigen binding to a B cell and can be used as a rough surrogate for binding affinity (79, 81, 82). Pre-incubation of B cells with increasing concentrations of a monomeric antigen prior to labeling with tetrameric antigen can also be used to further quantify binding affinity. Cells expressing high affinity BCRs will bind monomeric antigen at low concentrations, whereas low affinity BCRs will require higher concentrations of monomeric antigen to compete with and inhibit tetramer binding (26). Individual cells can also be isolated by fluorescence activated cell sorting (FACS) for downstream analysis, including BCR sequencing and cloning, BCR affinity measurement, *in vitro* proliferation, and transcriptional profiling.

## Methods for Rare Antigen-Specific B Cells

**Figure 1:**
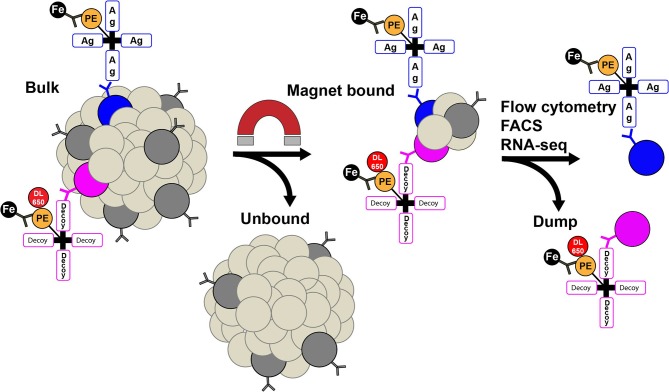
Rare antigen (Ag)-specific B cells can be identified using tetramers conjugated to a fluorochrome, followed by magnetic (Fe) nanoparticles that bind the fluorochrome, magnetic enrichment, and flow cytometry. B cells are shown in dark gray with a B cell receptor on the surface. PE, phycoerythrin; DL650, DyLight 650; FACS, fluorescent activated cell sorting.

Methods have recently been developed to further improve the sensitivity for detecting rare antigen-specific B cells. Magnetic nanoparticles conjugated to antibodies targeting the fluorochrome on the antigen of interest, allow for the enrichment of antigen-specific B cells prior to flow cytometry (20, 26, 80, 83). This approach is particularly useful for detecting rare antigen-specific naïve B cells, autoreactive B cells, memory B cells, and plasmablasts (21, 26, 47, 50). The magnetic enrichment strategy allows for the analysis of significantly more cells in a shorter period of time by concentrating the cells of interest prior to flow cytometry (Figure 1). Notably, as with any method that seeks to identify a population of cells at a very low frequency, the background and noise inherent in the detection system is magnified with respect to the signal of interest, especially when that signal is weak. Therefore, to detect the antigen-specific population of interest, the following considerations are critical: (1) Using decoys to exclude B cells of unwanted specificities; (2) careful design of flow cytometry panels to avoid emission spillover into the channel for the antigen of interest; and (3) choosing the brightest fluorochromes, like R-phycoerythrin or allophycocyanin.

## *In vivo* Assays for Studying Antigen-Specific Primary B Cell Responses

*In vivo* methods to probe antigen-specific B cell responses in the presence of other antigen-presenting cells and T cell helpers, have increased our mechanistic understanding of the humoral immune response during vaccination, infection, and autoimmunity. Adoptively transferred B cells can be distinguished from recipient lymphocytes by taking advantage of mouse strains with allelic variations in CD45 or mice devoid of B cells. The adoptively transferred B cells can come from wild-type mice or from mice expressing transgenic BCRs (Table 2), and antigen-specific B cells can be analyzed using the techniques described above.

**Table 2:** Examples of B cell receptor transgenic mice.

**Antigen**	**Disease or model**	**References**
**MODEL ANTIGENS**		
Hen egg lysozyme	B cell selection	(84)
4-hydroxy-3-nitrophenylacetyl	B cell selection	(85, 86)
Trinitrophenyl	B cell selection	(87)
Ovalbumin	B cell selection	(88)
Phosphocholine	B cell selection	(89, 90)
H-2K (MHCI)	B cell selection	(91)
Chicken gamma globulin	B cell selection	(92)
**AUTOIMMUNE DISEASES**		
Red blood cells	Autoimmune hemolytic anemia	(93)
Single stranded DNA	Systemic lupus erythematosus	(94)
Double stranded DNA	Systemic lupus erythematosus	(95)
Rheumatoid factor	Systemic lupus erythematosus	(96, 97)
Myelin oligodendrocyte glycoprotein	Multiple sclerosis	(98)
Insulin	Type I diabetes	(82, 99)
**INFECTIOUS DISEASES**		
Envelope	HIV	(72, 100–103)

Microscopy is another general technique that has been used to identify antigen-specific cells *in vivo* and offers the advantage of direct visualization. In the first reported application of this technique to demonstrate the cellular origin of antibodies in 1955, fluorescein-conjugated antibodies against ovalbumin and human immunoglobulin were used to stain tissue sections of the spleen from hyperimmune rabbits (2). Since then, other groups have fluorescently labeled antigens to localize antigen-specific B cells by microscopy (62, 65). Advances in laser capture dissection microscopy, already used in the T cell field, also provide an opportunity for isolating individual antigen-specific B cells for downstream analysis, including sequencing and cloning of the BCR or transcriptional profiling (66). However, antigen staining of BCRs *in situ* can be challenging depending on the binding of antigens from pathogens to other cellular receptors or an alteration of BCR specificity during tissue fixation or processing. Two-photon or multiphoton microscopy has the ability to resolve images at greater depths and with less photobleaching than confocal microscopy (67, 68). As a result, this technology has allowed real-time imaging in living, intact lymphoid tissues of mice, permitting the direct *in vivo* observation of immune cell interactions. The dynamic movements and interactions of antigen-specific B cells can be studied *in vivo* by combining an adoptive transfer of individual B cells (isolated by limiting dilution or FACS) with two-photon microscopy (63, 69, 70).

## Emerging Techniques for Studying Antigen-Specific Primary B Cell Responses

Humanized mouse models are powerful tools for translating experiments in mice to applications in humans. Transgenic mice that produce humanized cytokines by knock-in replacement can be used to support human hematopoietic stem cells (104). Transgenic mice with complete humanization of the mouse immunoglobulin loci provide an opportunity for recapitulating the breadth of the human B cell repertoire and serve as a valuable tool for therapeutic antibody discovery (71). However, one caveat is that the allele frequencies found in the B cell repertoires of these mouse models may not necessarily recapitulate those found in humans (72).

Mass cytometry has the potential to provide further high-dimensional analysis of antigen-specific B cells. In this method, heavy metal ion tags rather than fluorochromes are used to label cells. Since data is collected as time-of-flight mass spectrometry, up to 42 unique parameters can be simultaneously measured from a single sample without significant spillover between channels or the need for compensation. Mass cytometry with heavy metal-labeled tetramers can be constructed using streptavidin (73). Mass cytometry with metal-labeled peptide-MHC tetramers has been used successfully to identify and characterize antigen-specific T cells, but to our knowledge has not yet been applied to antigen-specific B cells (73, 74). One limitation of this approach is that cells are unavailable for downstream analysis since they are vaporized by a plasma torch to atomize the ion tags. However, by simultaneously detecting many more surface markers and intracellular cytokines, transcription factors, and detecting more signaling molecules from individual cells than previously possible with traditional fluorescent labels, the application of mass cytometry with dimensionality reduction algorithms could help dissect the complexity of the B cell compartment, provide a higher resolution view of B cell development, and reveal novel subsets of antigen-specific B cells involved in mediating autoimmune diseases or protection against infection.

On the horizon, single cell RNA-sequencing (RNA-seq) technologies have the potential to revolutionize the study of antigen-specific immune cells (75, 76). The ability to generate a library of tetramers with unique barcodes could allow the simultaneous examination of gene expression profiles from a large number of cells with different antigen specificities in a single experiment. Combining barcoded tetramers with oligonucleotide-conjugated antibodies and RNA-seq to simultaneously measure the protein and gene expression of antigen-specific cells could further increase the amount of unbiased multi-omic information about individual antigen-specific cells in normal and disease states and aid the rational design of vaccines and therapeutics (105–107).

## Conclusions and Horizons

The ongoing analysis of antigen-specific B cell responses has led to the development of new diagnostic, therapeutic, and research reagents. Methods for studying antigen-specific B cell responses are being increasingly applied to tackle diseases like HIV, RSV, and autoimmune diseases, in which the immune response either fails to protect or clear disease, or where it enhances disease or is responsible for the disease itself. Considerable opportunities exist on the horizon for applying these methods to a myriad of diseases in which B cells play an active role.

## Author Contributions

JB and JT reviewed the literature, generated figures and tables, and wrote the manuscript.

### Conflict of Interest Statement

The authors declare that the research was conducted in the absence of any commercial or financial relationships that could be construed as a potential conflict of interest.
